# Supplementary material for: Genome-Scale Characterization of Predicted Plastid-Targeted Proteomes in Higher Plants
Source: Sci Rep. 2020 May 19;10:8281. doi: 10.1038/s41598-020-64670-5 (PMC7237471; doi:10.1038/s41598-020-64670-5)
Supplement: Supplementary file 1 [file 41598_2020_64670_MOESM1_ESM.zip › Supplementary File 1/Supplementary File 1_ReadMe.docx]

Supplementary File 1

- Supplementary file 1.1: This spreadsheet presents the sensitivity, specificity, MCC, and ACC calculations for each individual program and each combination of programs.
  - Tab 1 – combined: contains all sequences from PPDB, AtCHLORO, CropPAL, CropPAL2, and SUBA4, filtered for redundant sequences
  - Tab 2—experimental: contains only sequences with either GFP or MS experimental validation from above databases.
  - Tab 3—GFP Combined: contains only sequences with GFP experimental validation from above databases.
  - Tab 4—MS Combined: contains only sequences with MS experimental validation from above databases.
  - Tab 5—GFP+MS Combined: contains only sequences with both GFP and MS experimental evidence, and with agreement in subcellular location from above databases.
- Supplementary file 1.2: Contains detailed subcellular prediction outputs for Nonplastidial sequences, as defined by predictions from PPDB, AtCHLORO, CropPAL, CropPAL2, and SUBA4. Contains binary calculations for “accept/reject” as nonplastidial.
  - Tab1—Combined: detailed results for all nonplastidial sequences as defined by above databases. Contains any result not defined as chloroplast, plastid, or other cognates.
  - Tab 2—Experimental: detailed results for nonplastidial sequences as defined by above databases with either GFP or MS experimental evidence.
  - Tab 3—GFP combined: detailed results for nonplastidial sequences as defined by above databases with GFP experimental evidence.
  - Tab 4—MS combined: detailed results for nonplastidial sequences as defined by above databases with MS experimental evidence
  - Tab 5—GFP+MS combined: detailed results for nonplastidial sequences as defined by above databases with both MS and GFP experimental evidence, and with agreement in subcellular location.
- Supplementary file 1.3: Contains detailed subcellular prediction outputs for plastidial sequences
  - Tab1—Combined: detailed results for all plastidial sequences as defined by above databases. Contains any result defined as chloroplast, plastid, or other cognates.
  - Tab 2—Experimental: detailed results for plastidial sequences as defined by above databases with either GFP or MS experimental evidence.
  - Tab 3—GFP combined: detailed results for plastidial sequences as defined by above databases with GFP experimental evidence.
  - Tab 4—MS combined: detailed results for plastidial sequences as defined by above databases with MS experimental evidence
  - Tab 5—GFP+MS combined: detailed results for plastidial sequences as defined by above databases with both MS and GFP experimental evidence, and with agreement in subcellular location.
- Supplementary file 1.4: Same as Supplementary file 1.1, but with WoLF PSORT removed.
