# Supplementary material for: Genome-Scale Characterization of Predicted Plastid-Targeted Proteomes in Higher Plants
Source: Sci Rep. 2020 May 19;10:8281. doi: 10.1038/s41598-020-64670-5 (PMC7237471; doi:10.1038/s41598-020-64670-5)
Supplement: Supplementary file 2 [file 41598_2020_64670_MOESM2_ESM.pdf]

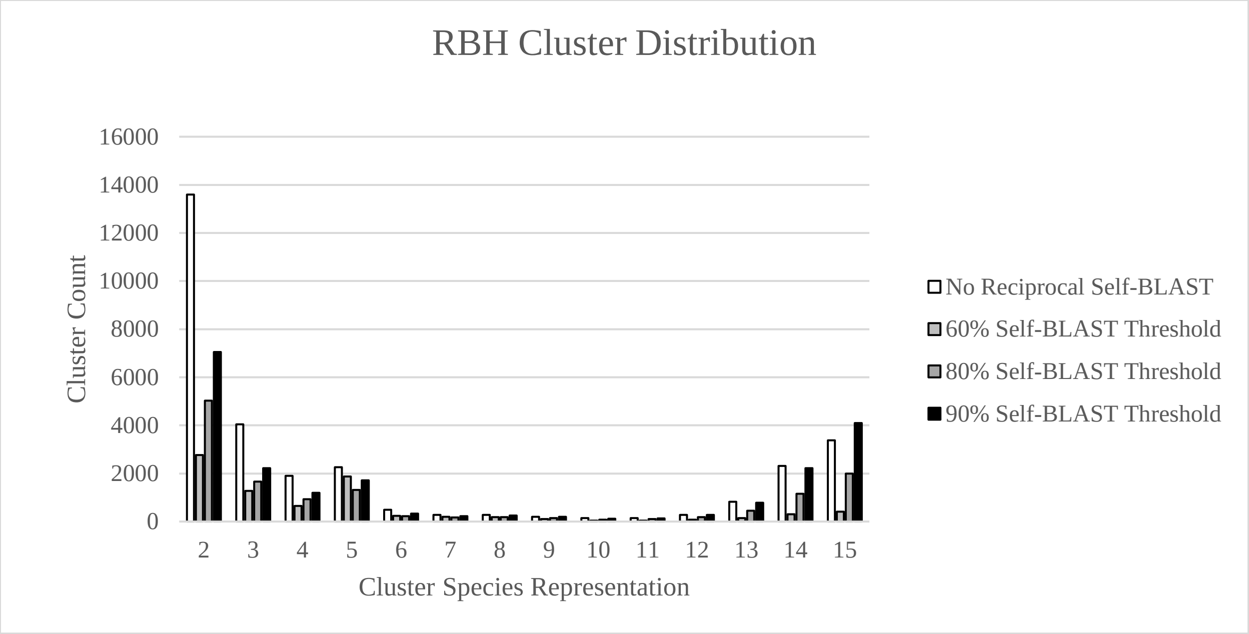

Supplementary Figure 2-1A: Reciprocal Best Blast Cluster Distribution by Species Representation. The number of clusters detected for unexpanded RBH methods without inclusion of better reciprocal hits within single species' proteomes is compared to three different expansion thresholds. Inclusion of any level of cluster expansion dramatically decreases the number of small clusters, but looser thresholds also cause decreases in the number of large clusters, indicating that inaccurate merging of multiple clusters into paralogous or nonhomologous large clusters is occurring. A 90% threshold for inclusion of reciprocal better hits within each proteome was determined to be the optimal method for supplementing best reciprocal hits between proteomes. Singleton and single species clusters not shown due to graph skew.

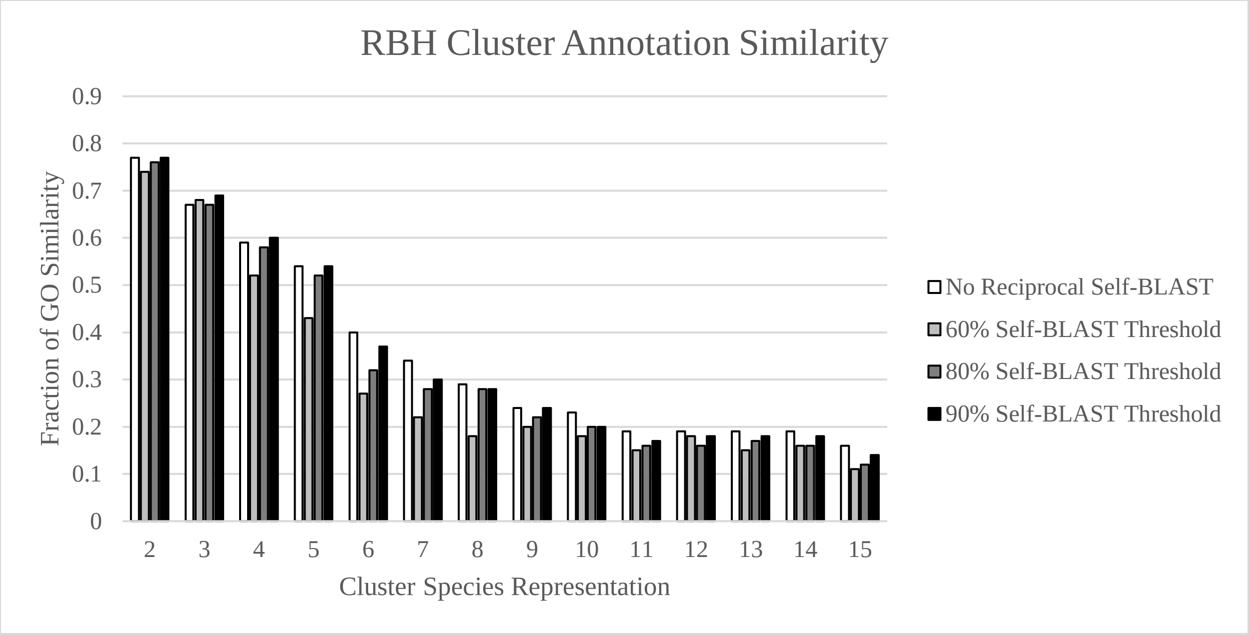

Supplementary Figure 2-1B: Reciprocal Best Blast Cluster Distribution by Species Representation. Inclusion of paralogous or nonhomologous sequences in clusters can be expressed as the fraction of sequences sharing common GO terms. This figure demonstrates that more lenient cluster expansion techniques cause loss of GO similarity within clusters compared with unexpanded clusters where no reciprocal self-BLAST hits are included. GO similarity was found to decrease as the number of species in a cluster increased. This is partially due to differing annotation methods used for different genome projects, but may also indicate low homology.

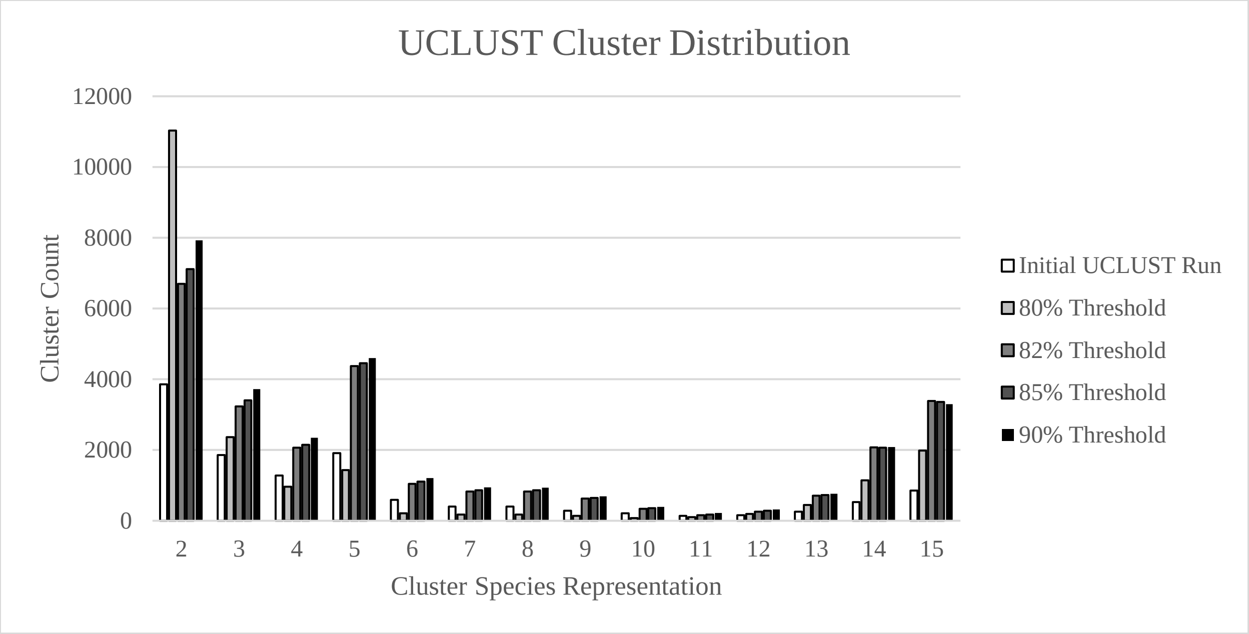

Supplementary Figure 2-2A: UCLUST Cluster Distribution by Species Representation. The number of clusters detected for unexpanded UCLUST methods without implementation of randomized runs to expand clusters is compared to four thresholds of expansion. All expansion methods lead to increases in cluster sizes, mostly as a result of reducing the number of singleton and single-species clusters (not shown due to graph skew).

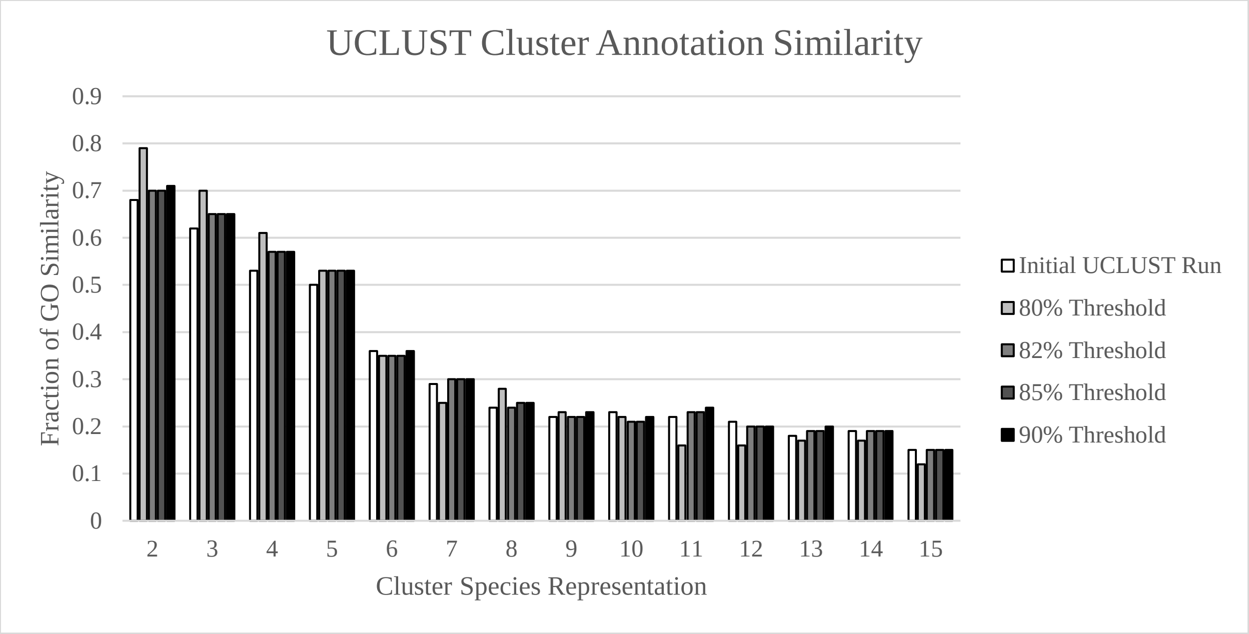

Supplementary Figure 2-2B: UCLUST Cluster Distribution by Species Representation. As with RBH methods, GO similarity was tested within clusters to determine how four different thresholds of cluster expansion affected homology. Expansion at all levels led to increased GO similarity at smaller cluster sizes, while all expansion thresholds except 80% had similar levels of similarity at larger cluster sizes.

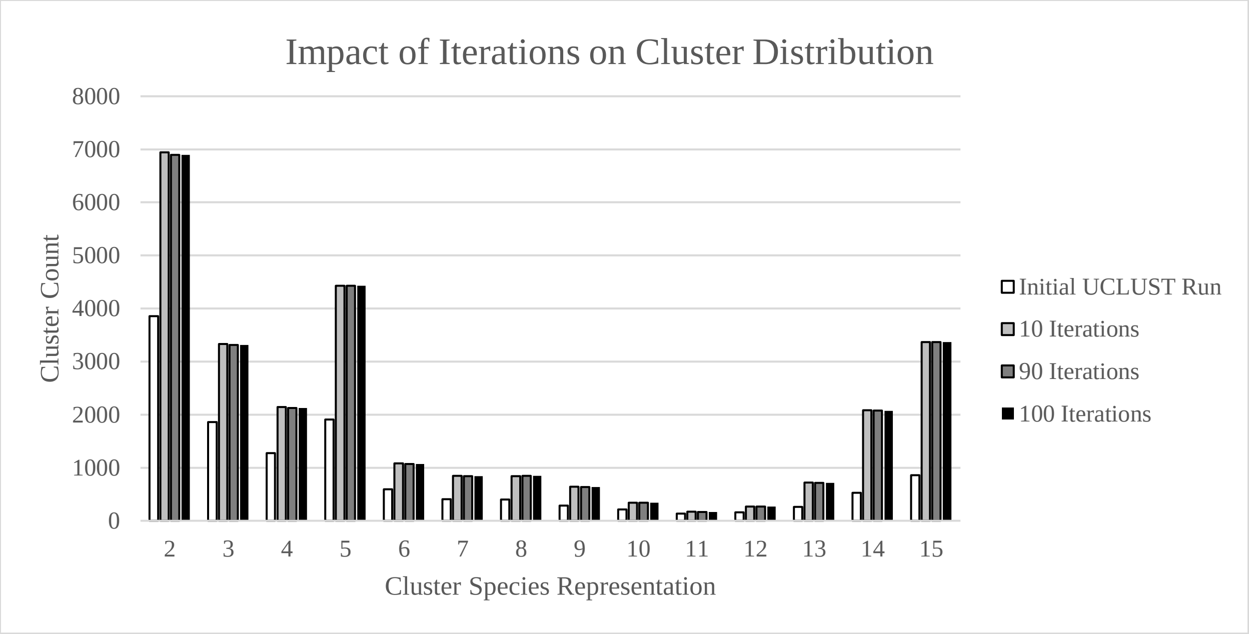

Supplementary Figure 2-3A: Impact of Iteration Number on UCLUST Cluster Expansion. A large shift between the initial UCLUST run and the 10-iterations experiment shows that the majority of cluster space is captured, with very slight differences between the 10-iterations experiment and 90 or 100 iterations. Addition of random seeding causes shifts from monospecies clusters towards multi-species clusters at all levels. Monospecies clusters are not shown due to graph skew.

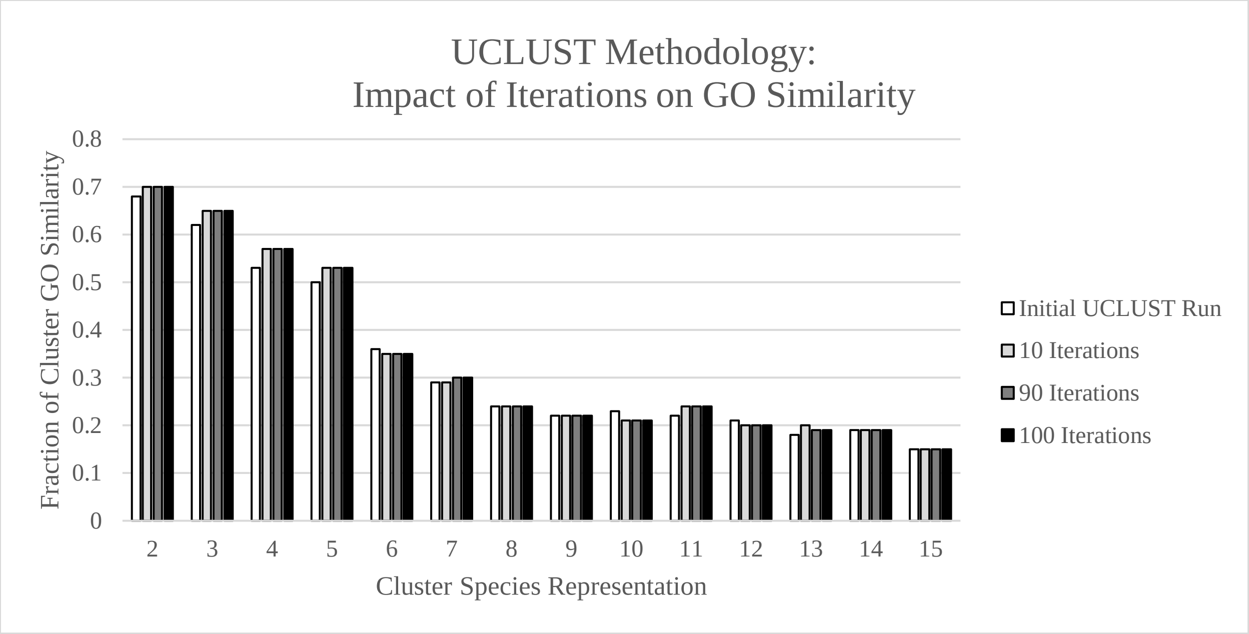

Supplementary Figure 2-3B: Impact of UCLUST Expansion Iterations on GO Similarity. GO annotation was largely stable across all multispecies clusters, with slight increases observed for smaller clusters and for clusters containing 7, 11, and 13 species. Clusters with 6 or 12 species showed slight decreases, while all other clusters remained stable. Monospecies clusters are not represented due to the high number of singleton sequences.
