# Supplementary material for: Genome-Scale Characterization of Predicted Plastid-Targeted Proteomes in Higher Plants
Source: Sci Rep. 2020 May 19;10:8281. doi: 10.1038/s41598-020-64670-5 (PMC7237471; doi:10.1038/s41598-020-64670-5)
Supplement: Supplementary file 3 [file 41598_2020_64670_MOESM3_ESM.zip › Supplementary File 3/Supplementary File 3_ReadMe.docx]

Supplementary File 3: Detailed results of RBH clustering method. For each tab, “Total Proteome” refers to all detected sequences within the species’ full published protein dataset, while “Plastome” refers to only the subset of those sequences with predicted chloroplast targeting as defined by consensus between “TargetP” and “Localizer”. See materials and methods for specific parameters used in each tab.

- Tab 1—Plastidial: Full list of clusters with at least 1 plastidial sequence, with number of sequences per species per cluster, and a full list of the sequence aliases.
- Tab 2—Shared: Full list of clusters with at least 12 species, with number of sequences per species per cluster, and a full list of the sequence aliases.
- Tab 3—Shared-synonyms: Contains a list of protein names for each detected sequence from Arabidopsis from clusters listed in Tab 2.
- Tab 4—Shared-Arabidopsis: contains a list of protein sequence ID’s for Arabidopsis from each cluster from Tab 2. Used to generate gene names listed in Tab 3.
- Tab 5—Unique: Full list of clusters containing sequences from at least 3 species of which only 1 has a chloroplast transit peptide.
- Tab 6—Monocots: Full list of clusters in which only Monocot sequences, but no Dicot sequences, had chloroplast transit peptides.
- Tab 7—Poaceae: Full list of clusters in which only Poaceae sequences, but no other Monocot or Dicot sequences, had chloroplast transit peptides.
- Tab 8—Dicots: Full list of clusters in which only Dicot sequences, but no monocot sequences, had chloroplast transit peptides
- Tab 9—Rosids: Full list of clusters in which only Rosid sequences, but no other Dicots or Monocots, had chloroplast transit peptides.
- Tab 10—Rosaceae: Full list of clusters in which only Rosaceae sequences, but no other Dicots or Monocots, had chloroplast transit peptides.
