# Supplementary material for: Genome-Scale Characterization of Predicted Plastid-Targeted Proteomes in Higher Plants
Source: Sci Rep. 2020 May 19;10:8281. doi: 10.1038/s41598-020-64670-5 (PMC7237471; doi:10.1038/s41598-020-64670-5)
Supplement: Supplementary file 5 [file 41598_2020_64670_MOESM5_ESM.zip › Supplementary File 5/Supplementary File 5_ReadMe.docx]

Supplementary File 5:

Detailed GO term results, TargetP results, Localizer results, and cluster name for RBH (BLAST) and UCLUST methods for each sequence, separated by species. Final four columns of each document list whether the sequence was identified as a species-unique chloroplast-targeted sequence, and whether it was detected as such by RBH (BLAST), UCLUST, or both methods.

- Column A: Sequence ID as defined by the associated publication which published that species’ genome and proteome.
- Column B: Sequence name- “Alias” assigned for the purposes of this publication.
- Column C: Alt GO ID’s: primary GO terms identified using BLAST2GO
- Column D: simplified GOslim format for GO terms in Column C.
- Column E: Localizer prediction (chloroplast column only)
- Column F: TargetP Prediction
- Column G: Consensus Prediction- binary report of whether chloroplast targeting was detected in both TargetP and Localizer. Sequences with “1” were defined as “Plastidial,” and sequences with “0” were defined as “Nonplastidial” for this publication.
- Column H: BLAST Cluster: name of RBH (BLAST) cluster to which the sequence mapped
- Column I: UCLUST Cluster: name of UCLUST cluster to which to sequence mapped
- Column J: BLAST-Only: if the sequence was found to be a species-unique chloroplast targeted protein only via RBH (BLAST) methods, the cluster ID is listed here.
- Column K: UCLUST-Only: if the sequence was found to be a species-unique chloroplast targeted protein only via UCLUST methods, the cluster ID is listed here.
- Column L: Consensus: if the sequence was found to be a species-unique chloroplast targeted protein by both BLAST (RBH) and UCLUST methods, the cluster ID is listed here.
- Column M: Unique: demarcates whether the sequence was found to be a species-unique chloroplast targeted protein by any method.
