# Supplementary material for: Genome-Scale Characterization of Predicted Plastid-Targeted Proteomes in Higher Plants
Source: Sci Rep. 2020 May 19;10:8281. doi: 10.1038/s41598-020-64670-5 (PMC7237471; doi:10.1038/s41598-020-64670-5)
Supplement: Supplementary file 6 [file 41598_2020_64670_MOESM6_ESM.zip › Supplementary File 6/Supplementary File 6_ReadMe.docx]

Supplementary File 6: Detailed results for *de novo* transcriptome and proteome identification in *Malus* x *domestica*, as described in the materials and methods section

- Tab 1—Transcriptome: RNA sequence of each identified ORF with >300bp and with at least 5x coverage.
- Tab 2—5x_TranslatedProteome: Translated protein sequence from each ORF in Tab 1.
- Tab 3—BLAST-Velasco: Translated ORF’s with best identified BlastP hits to the proteome published by Velasco et al., 2009.
- Tab 4—Headers: for each translated protein sequence from the *de novo* transcriptome assembly, the final alias refers to the sequence ID used for this publication, and the column C “MDP Designation” refers to whether the sequence was identified as likely to be the same as a sequence reported in Velasco et al., 2010.

Velasco R, Zharkikh A, Affourtit J, Dhingra A, Cestaro A, Kalyanaraman A, et al. The genome of the domesticated apple (Malus × domestica Borkh.). Nat Genet. 2010;42(10):833-9. doi: 10.1038/ng.654.
